# External Beam Radiotherapy in the Management of Uveal Melanoma

**DOI:** 10.1007/s11864-024-01212-5

**Published:** 2024-06-13

**Authors:** Melek Tugce Yilmaz, Sezin Yuce Sari, Faruk Zorlu, Gozde Yazici

**Affiliations:** Hacettepe University Faculty of Medicine, Department of Radiation Oncology, Ankara, Turkey

**Keywords:** Uveal melanoma, External radiotherapy, Protons, Stereotactic radiosurgery

## Abstract

Uveal melanoma is the most common primary ocular tumor in adults. With the evidence demonstrating that episcleral plaque brachytherapy (EPB) has similar survival rates as enucleation in the Collaborative Ocular Melanoma Study (COMS), eye-sparing treatments have come to the fore today. External radiotherapy techniques (proton beam radiotherapy and stereotactic radiosurgery/fractionated stereotactic radiosurgery) are an important treatment option for globe-sparing treatments. There are no prospective randomized trials comparing these techniques; however, retrospective series, meta-analyses, and reviews indicate that these EPB and external radiotherapy techniques are equal. With this review, we aimed to examine the external radiotherapy techniques used in the treatment of uveal melanoma in detail with reference to the current literature.

## Introduction

**Fig. 1.**
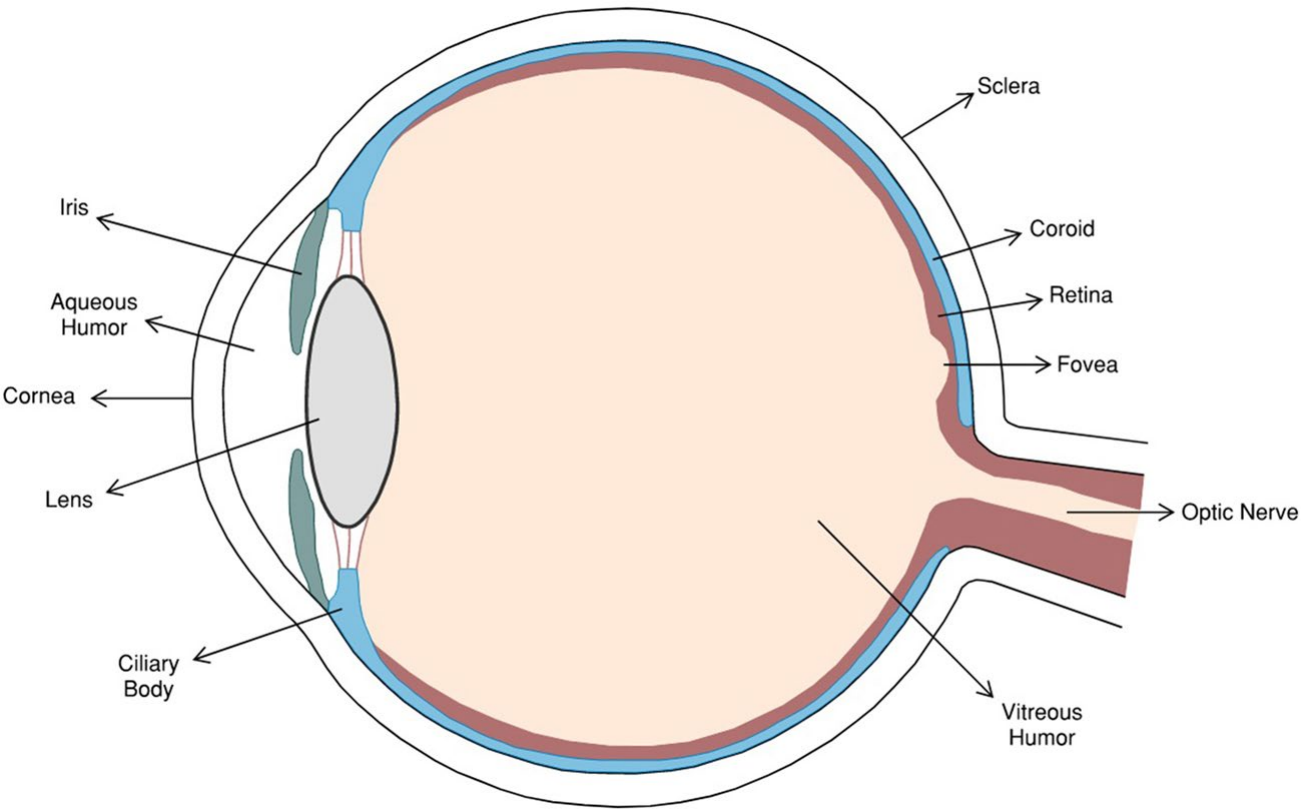
Illustration depicting the anatomy of an eye.

Uveal melanoma (UM) is the most common primary eye tumor in the adult population [1]. The uvea is made up of three main structures: the iris, ciliary body, and choroid, yet 90% of the UM develop from the choroid (Fig. 1) [2]. Although UM is a relatively rare tumor, more than half of patients develop distant metastases, and in 90% the site of metastasis is the liver [3–5]. Only 2–3% of patients with UM had distant metastases at the time of diagnosis, and the chances of survival following metastasis are quite low [6–8]. Therefore, local treatments at the time of diagnosis are crucial for patient survival.

Enucleation was the standard of care in the local treatment of UM until the 1970s. Retrospective studies indicating that enucleation and radiotherapy (RT) may have similar survival paved the way for the Collaborative Ocular Melanoma Study (COMS) prospective randomized trial comparing enucleation and Iodine-125 (^125^I) episcleral plaque brachytherapy (EPB) [9–12]. The promise of RT to provide local control by protecting both the eye and vision has made it the most frequently used treatment modality today [13].

The RT technique most frequently used to treat UM today is EPB. ^125^I is the most frequently used isotope. Besides ^125^I, other isotopes including Cobalt-60 (^60^Co), Iridium-192 (^192^Ir), Palladium-103 (^103^Pd), and Ruthenium-106/Rhodium-106 (^106^Ru/^106^Rh) are also employed [14–18]. The American Brachytherapy Society (ABS) guidelines recommend treatment with EPB without biopsy in patients with clinically growing UM [19]. According to ABS, EPB is not recommended for tumors with T4e extraocular extension, basal diameter that exceed the limitations of brachytherapy, blind sore eyes, or eyes with no light perception vision.

Apart from EPB, proton beam RT (PBRT) and stereotactic radiosurgery/fractionated stereotactic radiosurgery (SRS/FSRS) are alternative globe-sparing RT techniques. Although there is no prospective randomized trial proving the efficacy of SRS/FSRS and PBRT, reviews and meta-analysis indicate similar survival rates [20–22, 23••]. In particular, the invasive nature of EPB, high radioactive source prices, the need for trained dedicated personnel for brachytherapy application, significant learning curve, and difficulties in use -especially in tumors close to the optic nerve- limit its widespread use [24]. In this review, external RT techniques used as an alternative to EPB will be discussed.

### Protons

Proton beam RT is the most preferred RT modality after plaque brachytherapy in eye sparing approach. The most prevalent strategy nowadays is a single beam, with the dose adjusted to the target utilizing single scattering and Bragg peak [25•]. For precise tumor targeting, radiologic spotting with tantalum markers is frequently employed in treatment planning. Light-field set-up is a method used in anteriorly located tumors [26]. Pencil beam − based techniques such as uniform and modulated scanning are also used in treatment planning [27, 28].

PBRT is the treatment modality with the most experience in external RT techniques of UM and its earliest usage dates back to the 1970s [29]. PBRT was shown to have 5-year local control rates of 93.9%-96.5% and ocular protection rates of 71.3%-95% in large series (Table 1) [30–37]. Despite the lack of prospective randomized trials comparing PBRT with traditional EPB, reviews and meta-analyses show that the two modalities have comparable rates of survival, local control, and eye protection [21, 38]. Moreover, in a prospective randomized study comparing EPB and helium ions, increased local control, disease-free survival (DFS) and eye preservation were obtained with particle RT [39]. In the University of San Francisco (UCSF) experience reported by Char et al., ^125^I EPB and charged particle therapy were compared and particle RT was reported as having greater local control with a 96.5% rate [40].

**Table 1:** Large series reported proton beam radiotherapy outcomes in uveal melanoma with at least 100 patients

Publication	Period	N	Dose	Median Follow up	LC	Eye preservation	OS	Eye fixation	Toxicity
Egger et al. 2003 (Switzerland) (30)	1984–1999	2648	60 Gy RBE in 5 fractions	24 months	5 years 95.8 10 years 94.8	91.8%	10 year 73%	Fixed gaze with target light	-
Damato et al. 2005 (Liverpool-Clatterbridge) (31)	1993–2003	349	48.3 Gy RBE in 3 fractions	37 months	5 year 96.5%	92%	Death from metastatic disease 2.5% at 2 year, 10% at 5 year, 14.1% at 8 year	Local anesthetic drops	17.9% glaucoma 12.8% rubeosis 16.7% pain 9% vitreous bleeding 38% retinal detachment
Dendale et al. 2006 (France Curie-Orsay) (32)	1991–2001	1406	60 Gy RBE in 4 fractions	73 months	5 year 96%	-	5 year 79%	Fixed gaze with target light	66.5% maculopathy 23.4% papillopathy 28.6% glaucoma 61.8% cataract 11.5% keratitis 13.9% vitreous hemorrhage 27.5% intraocular inflammation
Caujolle et al. 2010 (France-Nice) (33)	1991–2007	886	60 Gy RBE in 4 fractions	64 months	5 year 93.9%, 10 year 92.1%	92.2%	5 year 79.4% 10 years 64.1% 15 year 54.2%	Fixed gaze with target light	17% glaucoma 27.5% retinopathy 31.7% cataract 7.8% optic neuropathy
Macdonald et al. 2011 (Scotland) (34)	1993–2008	147	58 Gy RBE in 4 fractions	53 months	-	5 year 71.3%	24.5% all-cause mortality	-	
Mishra et al. 2013 (UCSF) (35)	1996–2010	704	56 Gy RBE in 4 fractions	58 months	-	-	-	-	4.9% enucleation due to neovascular glaucoma
Lane et al. 2015 (Harvard) (36)	1975–2005	3088	70 Gy or 50 Gy RBE in 5 fractions	148 months	-	-	Melanoma related mortality 15 years 24.6% 20 years 25.8% 25 years 26.4%	-	-
Seibel et al. 2015 (Berlin) (37)	1998–2008	982	60 Gy RBE in 4 fractions	60.7 months	1 year 98% 3 years 97% 5 years 96% 10 years 94%	95%	1 year 99% 3 years 90% 5 years 80% 10 years 60%	-	12.1% neovascular glaucoma

Abbreviations: *N* Number; *LC* Local control; *OS* Overall survival

The first series using PBRT in UM was belonged to the Harvard group and reported by Gragoudas et al. in 1980 [29]. In the study, 7000–9000 cobalt rad equivalent doses were used in 36 patients. Half of the patients had medium-sized and 42% had large-sized tumors. Enucleation rate at first year was 27% with an average 16-months of follow-up [36, 41]. The same group reported the results of 3008 patients treated between 1975 and 2005 in 2015. In this study melanoma-related mortality was reported as 24.6%, 25.8%, and 26.4% for 15, 20, and 25 years, respectively. Although a dose of 70 Gy RBE (relative biological effectiveness) was used in this study, the same group's prospective randomized trial revealed that a dose of 50 Gy RBE produced equivalent survival and local control outcomes with improved visual field preservation [42]. In a further trial that only included large-sized tumors, the Harvard group reported that the eye retention rate at 1, 5, and 10 years was 95.1%, 77.4%, and 70.4%, respectively [43].

The Swiss group reported one of the largest series in the literature in 2001 and 2003 [30, 44]. In 2435 patients with a median follow-up of 40 months, 5-year local control was 95.8% and 10-year local control was 94.8%. Variables affecting local control were: ciliary body tumors with large size, reduced margins, improper tantalum clip location, eyelids inside the treatment area, and male gender. They divide the treatment periods into: before 1988, 1988–1993, and after 1993, and found that patients treated after 1993 had better local control. This was ascribed to advancements in the implantation method. In their 2003 report, on the other hand, they emphasized on eye retention outcomes [30]. At 5, 10 and 15 years, eye retention rates were 88.9%, 86.2% and 83.7%, respectively. Enucleation was found to be associated with tumor size, specifically tumor height, tumor location, gender, intraocular pressure, and a retinal detachment status at the time of therapy. Neovascular glaucoma was found in 25.3% of cases. Their results demonstrates that even in tumors that are thought to be unfavorable for brachytherapy and enucleation is inevitable, eye protection was feasible.

Dendale et al. reported comparable results in 1406 patients treated with a dose of 60 Gy RBE in 4 fractions. They achieved a 96% local control rate and 79% overall survival (OS) rate [32]. The side effect profile was reported in detail, and the rates were 66.5% for maculopathy, 23.4% for papillopathy, 28.6% for glaucoma, 61.8% for cataract, 11.5% for keratitis, 13.9% for vitreous hemorrhage, 27.5% for intraocular inflammation in median 73 months follow-up.

Damato et al. published their experience in iris and choroidal melanoma in two separate reports in 2005 [31, 45]. They included 349 patients in their choroidal melanoma report, and 48.3 Gy RBE was applied in 3 fractions [31]. At a median follow-up of 37 months, 5-year local control rate was 96.5% and overall eye preservation rate was 92%. Death from metastatic disease was 2.5%, 10% and 14.1% at 2, 5 and 8 years respectively. The outcomes of iris melanoma was reported in 88 patients [45]. In this report, which was focused on the side effects, they reported that 24% of the patients who did not initially have cataracts developed cataracts secondary to RT, and 4% of the patients who did not initially have glaucoma developed secondary neovascular glaucoma, and they concluded that PBRT was well tolerated in iris melanoma.

The outcomes of PBRT in iris melanoma have been addressed by Thairat, Oxenreiter, and Gollrad et al. They observed local recurrence rates ranging from 2.2% to 9.6% for doses of 60, 70, and 50 Gy RBE, respectively. They reported secondary neovascular glaucoma ranging from 7.6% to 16.8% in circumscribed tumors and 11.8%-71.9% in diffuse tumors. With its acceptable toxicity and significant ocular retention rates, PBRT remains to be a viable therapy approach, particularly in circumscribed tumors [26, 46, 47].

### Gamma-knife Based SRS/FSRS

Photon based SRS/FSRS experiences started with Gamma Knife in the early 90 s and the first reports are from the early 2000s. The studies reported 84–98% local control and 75–94% eye preservation rates (Table 2) [48–55].

**Table 2:** Series reported gamma-knife based SRS/FSRS outcomes in uveal melanoma with at least 50 patients

Publication	Period	N	Dose	Median Follow up	LC	Eye preservation	OS	Eye fixation	Toxicity
Mueller et al. 2000 (47)	1996–1999	58	25 Gy to 50% isodose	-	97%	94%	-	Retrobulbar anesthesia	66% retinal detachment 1% radiation retinopathy 1% iris neovascularization
Zehetmayer et al. 2000 (48)	1993–1997	62	2 × 35 Gy 2 × 30 Gy 2 × 25 Gy 3 × 15 Gy	28.3 months	98%	87%	-	Ocular suction system	43% lens opacity 20% neovascular glaucoma 22% retinopathy 25% optic neuropathy 12% corneal epithelial defects 8% vitreous hemorrhage
Simonova et al. 2002 (49)	1996–2001	81	1 × 20–76.5 Gy	-	84%	87%	70% at 15 months	Rectus muscle sutures	24% neovascular glaucoma 9% corneal damage 12% optic nerve damage 13% iris damage
Sarici and Pazarli 2012 (50)	2002–2010	50	1 × 30 Gy to the 50% isodose	40 months	90%	3 years 88% 5 years 82%	3 years 94% 5 years 86%	-	10% vitreous hemorrhage 32% cataracts 26% radiation maculopathy 16% optic neuropathy 26% radiation retinopathy
Dinca et al. 2012 (51)	1990–2010	157	1 × 35–70 Gy	63.5 months	-	For 35 Gy, 45 Gy and 50–70 Gy, respectively: 93.55%, 76.06%, 75%	5 years 64% for 35 Gy, 62.7% for 45 Gy, 63.6% for 50–70 Gy	Rectus muscle sutures	For 35 Gy, 45 Gy and 50–70 Gy, respectively: Retinopathy 25.81%, 35.21%, 41.67% Vitreous hemorrhage 12.9%, 12.68%, 12.5% Optic neuropathy 16.13%, 12.68%, 20.83% Glaucoma 8.06%, 19.72%, 20.83% Cataract 11.29%, 25.35%, 25% Retinal detachment 4.84%, 8.45%, 16.67% Blindness 31.48%, 83.72%, 82.35%
Wackernagel et al. 2013 (52)	1992–2010	189	1 × 25–80 Gy	39.5 months	94.4%	85.9%	-	Retrobulbar anesthesia and rectus muscle sutures	4% optic neuropathy 3% vitreous hemorrhage 8% persistent retinal detachment 5% radiation maculopathy 7% retinopathy
Suesskind et al. 2013 (53)	2003–2008	60	1 × 25 Gy	33.7 months	2 year 94% 3 years %85	78%	2 years 93% 3 years 91%	Retrobulbar anesthesia	24% opticopathy occurred 42% retinopathy 13% macula edema 6% keratopathy 20% cataracts 15% neovascular glaucoma
Modorati et al. 2019 (54)	1993–2018	194	1 × 35.8 ± 4.7 Gy at 50% isodose curve	57.6 months	93.3%	90.7%	-	-	41.2% lens opafication 27.3% neovascular glaucoma 34.5% radiation retinopathy 11.4% maculopathy 18.6% optic neuropathy

Abbreviations: *N* Number; *LC* Local control, *OS* Overall survival

There are retrospective and dosimetric studies comparing SRS with proton beam and EPB, which is accepted as standard of care [56–58]. In the dosimetric study of Weber et al., fixed proton horizontal beam (OPTIS) and intensity-modulated spot-scanning proton therapy (IMPT) and SRS were compared. Conformity of the treatment and ipsilateral organs at risk (OAR) protection were similar with proton and photon, and a more homogeneous dose distribution in the target volume was obtained with proton [56]. Sikuade et al., compared patients treated with PBRT and SRS retrospectively and showed that both modalities had similar local control, survival, and eye-sparing rates [57]. Van Beek et al. found comparable rates of local control or adverse effects for PBRT and SRS. The risk vitreous hemorrhage was more common with FSRS and was the sole difference between two modalities [59]. Another comparison included Ruthenium EPB with 2–3 fractions of Gamma knife FSRS and 5 fractions of linear accelerator (linac)-based FSRS arms [58]. At three-year follow-up, 70% flat scar was seen in patients treated with the ^106^Ruthenium106 EPB compared to 85% scarring in patients treated with the other two external RT techniques. Hence, faster tumor response was achieved with the Ruthenium EPB. However, it should be kept in mind that the patients included in the external RT arm are patients with COMS large tumors who are not suitable for brachytherapy.

The first large series using the Gamma Knife-based SRS was reported by Marchini and Mueller et al. [48, 60]. They reported local control and eye protection rates of 95% and 90%-94% respectively. However the follow-up time of these first series were relatively short and doses ranging from 38–70 Gy were employed.

In their phase I/II prospective trial, Zehetmayer et al. reported the outcomes of 62 patients with a median follow-up of 28.3 months [49]. They applied 45–70 Gy in 2–3 fractions as opposed to the current series, which uses 1 fraction due to stereotactic frame. As a result, they reported 98% local control rate and 87% eye preservation rate. They concluded that multi-fraction radiosurgery would be more favorable than SRS in terms of both tumor control and side effects, since high-dose volume irradiated with more than 10 Gy/fraction is associated with morbidity, and stated that they created the treatment protocol for today's linac based Vienna series [61•].

In a cohort of 84 patients, Simonova et al. reported 84% local control rate and 87% eye preservation rate. Neovascular glaucoma, which was present in 24% of patients, was shown to be related with treatment volumes of 1000 mm^3^ and higher [50]. They also recommended that > 40 Gy should be prescribed in compliance with dose limitations since doses over 40 Gy are positive prognostic factor. Doses of maximum 9 Gy applied to the optic nerve, 10 Gy to the lens, and 15 Gy to the cornea were associated with serious side effects.

Modorati et al. published the largest Gamma knife based SRS/FSRS series in the literature [55, 62]. They reported 93.3% local control rate, and 90.7% eye preservation rate with doses deescalated from 50 to 35 Gy. Toxicity rates were 41.2% for lens opacification, 27.3% for neovascular glaucoma, 34.5% for radiation retinopathy, 11.4% for maculopathy, and 18.6% for optic neuropathy.

In a group of 189 patients, Wackernagel et al. examined the risk factors for enucleation and revealed that tumor recurrence, advanced tumor stage, lower basal visual acuity, retinal detachment prior to treatment, and the prescription dose (25–30 Gy vs. 35–80 Gy) were all correlated. Along with the trends, this team has also decreased the dose over time. The authors claimed that despite its borderline significance, the lower dose did not impair the outcome and provided better eye sparing with fewer side effects [53].

Suesskind et al. investigated the effect of tumor resection on local control after SRS and obtained similar eye sparing rates with or w/o local resection. The results showed that local resection after SRS could improve local control and decrease side effects. However, 3 unexplained deaths after surgery led to discontinuation of the protocol, and this approach has not gained widespread use due to similar eye preservation rates [54].

### Linear Accelerator Based SRS/FSRS

Linac-based SRS/FSRS is a treatment method that has become popular in the treatment of UM especially in the last decade. Although PBRT seems to be at the forefront in the treatment of UM, linac based SRS/FSRS is easily accessible, more common, more affordable and less invasive than PBRT. There is no prospective randomized trial comparing PBRT and linac based SRS/FSRS, and it seems unlikely to be one in the future due to the scarcity of the disease.

In the literature, 83%-95.4% local control rates and 62%-94.6% overall eye preservation rates have been reported with linac based SRS/FSRS (Table 3) [61•, 63••, 64••, 65•, 66]. Although Linac based SRS/FRS techniques became widespread after Gamma knife, It was quickly acknowledged by the radiation oncology community, and many centers have ongoing short- and long-term follow-up studies [63••, 64••, 65•].

**Table 3:** Large series reported linear accelerator based SRS/FSRS outcomes in uveal melanoma

Publication	Period	N	Dose	Median Follow up	LC	Eye preservation	OS	Eye fixation	Toxicity
Schmelter et al. 2022 (Munich) (62)	2005–2019	594	1 × 18–22 Gy	54.7 months	87,5%	81.8%	82% 4.5 years	Retrobulbar anesthesia	36.8% retinal detachment 18.1% secondary neovascular glaucoma 12.7%radiation retinopathy
Yazici et al. 2022 (Ankara) (63)	2007–2019	443	3 × 20 Gy	74 months	83%	62%	2-, 5-, and 10-year OS rate was 97%, 87%, 71%,	Peribulbar anesthesia	36% radiation retinopathy 27% optic neuropathy 18% cataracts
Van Beek et al. 2022 (Rotterdam) (64)	1999–2014	189	5 × 10 Gy	92.9 months	92.2%	94.6%	-	Rotterdam eye fixation system with blinking light and camera	35.1% radiation retinopathy 23.8% maculopathy 12.4% optic neuropathy 20.1% vitreous hemorrhage 67.8% cataracts 20% neovascular glaucoma
Eidenberg et al. 2021 (Vienna) (60)	1997–2016	335	5 × 14 Gy 5 × 12 Gy 5 × 10 Gy	78.6 months	95.4%	79.7%	10 year 74.7%	Eye fixation with light source and camera	57% ischemic retinopathy 37% cataract 21% neovascular glaucoma
Krema et al. 2019-(Toronto) (65)	1998–2006	64	5 × 14 Gy	37 months	94%	-	-	Eye-monitoring device green light-emitting diode (LED) and camera	53% radiation cataracts 80% tumor vasculopathy 81% radiation retinopathy 64% optic neuropathy 42% neovascular glaucoma

Abbreviations: *N* Number; *LC*: Local control; *OS* Overall survival

The largest series reporting linac based SRS outcomes in the literature belongs to the Munich group [63••]. This group published the Cyberknife (CK)-based SRS series of 594 patients in 2022 after reporting the preliminary findings in 2006 and 2016 [67, 68]. They reported 87.5% local control rate and 81.8% eye preservation rate at a median follow-up of 54.7 months. They stated the factors affecting local control was treatment plan without contrast magnetic resonance image (MRI), prescription dose below 21 Gy, and marginal miss due to insufficient margin. Accordingly, they recommended that at least 21 Gy for treatments with linac based SRS. The toxicity rates were 36.8% for retinal detachment, 18.1% for secondary neovascular glaucoma, and 12.7% for radiation retinopathy were observed.

The largest linac based FSRS series on the other hand belongs to the Hacettepe group. Yazici et al. reported early results in 2018 and extended long-term results in 2022 [64••, 69]. Unlike the Munich group, most patients received FSRS. In this study, 30% of patients had COMS large tumors, and 83% overall local control rate and 62% eye preservation rate was reported at a median 74 months of follow-up. They observed increased eye preservation rate with the application of at least 45 Gy 3 fractions every other day.

Van Beek et al. also published the long-term results of the Rotterdam series in 2022 [65•]. In this series with the longest follow-up period in the literature, they reported excellent outcomes such as 92.2% local control and 94.6% eye preservation at a median follow-up of 92.9 months. Unlike other groups, they apply 50 Gy FSRS regimen in 5 fractions on consecutive days with the Rotterdam Eye Fixation system [70].

Eidenberg et al., reported 95.4% local control rate and 79.7% overall eye preservation rate at the doses gradually deescalated from 5 × 14 Gy to 5 × 10 Gy at a median 78.6-month follow-up [61•]. Comparative analysis of the arms receiving 10 Gy per fraction vs. > 10 Gy per fraction (12 Gy and 14 Gy) revealed that the two groups' rates of local control, eye retention, and metastasis-free survival were comparable. Interestingly, similar side-effect profiles have been reported between low and high doses. The Vienna group, contrary to Hacettepe and Munich groups reported that dose escalation did not contribute to tumor control.

### What Dose/Fractionation Should We Use?

There is no consensus for dose & fractionation for photon-based SRS/FSRS or proton therapy for uveal melanoma. Given the 50% risk of radiation-related complications and treatment-related vision loss, balancing optimal tumor response with toxicity seems essential [64••]. The literature on the dose–response relationship seems rather contradictory. Although there are studies showing that there is a dose–response relationship, there are also studies that indicate that dose de-escalation does not impair the results. Therefore, highly heterogeneous dose fractionation schemes have been used in published series.

Early studies for proton therapy used doses such as 70 Gy RBE. The prospective study of the Harvard team showed that there was no difference in the oncological outcomes of 50/60 Gy RBE and 70 Gy RBE, and recent studies have frequently used 50–60 Gy RBE doses in the treatment protocol (Table 1) [42]. Contrarily, in studies for the helium atom, a 13% increase in local recurrence was observed when 48 Gy was given in 4 fractions [71].

Dose fractionation schemes for SRS and FSRS are also quite heterogeneous. The Munich group reported that doses of 21 Gy and above increased local control in their patients treated with SRS [63••]. Yazici et al., similarly reported that local control increased with doses above 45 Gy in their series where they applied FSRS in 3 fractions [64••]. However, there were also opposite opinions. In the Vienna series, 3 different doses were recruited and no difference in local control and survival could be observed by de-escalating the dose from 5 × 14 Gy to 5 × 10 Gy [61•]. Langmann et al. also found that patients treated with a marginal dose of 52.1 Gy vs 41.5 Gy had similar tumor regression and unsurprisingly lower dose caused fewer side effects [72]. However, we should keep in mind that these series have retrospective nature and the fraction numbers are vary significantly.

Fractionation is also a matter of debate. Despite the Hacettepe team emphasizing that every other day treatments increase eye-sparing while giving > 45 Gy doses, especially in large tumors, the Vienna team applies the treatment for 5 consecutive days and reported similar side effects with the literature [61•, 64••].

### Toxicity

The lack of a standard dose fractionation scheme has resulted in reporting different toxicity rates across treatment modalities and at different doses. As well as the RT technique and dose, toxicity may vary depending on the tumor location and size [35].

Radiation retinopathy is one of the most common side effects of RT. It has been reported with varying frequency in the range of 12.7–81% in the SRS/FSRS series (Table 3). It represents an occlusive disease appearing in retinal vessels secondary to radiation [73]. In particular, retinopathy rates < 10% in the early gamma knife series are generally associated with a short follow-up period. Dinca et al., reported 25.81%, 35.21%, and 41.67% retinopathy at doses ranging from 35 Gy, 45 Gy, and 50–70 Gy, respectively, in their study emphasizing the toxicity and dose relationship. Retinopathy is reported with PBRT at a rate of 23%- 67%, similar to SRS/FSRS [38].

Neovascular glaucoma is another most common side effect. Especially in tumors located in the anterior compartment, it can be seen frequently. It is reported with a frequency of 8%-42% in SRS/FSRS series, and between 7–28.6% in PBRT series (Tables 1, 2 and 3). The incidence of glaucoma is going up to 71% in the iris melanoma series reported in the last decade [47]. Secondary neovascular glaucoma is an important side effect because it causes pain, visual loss and enucleation due to toxicity, and indirectly reduces ocular sparing rates.

Among the other frequently reported side effects, cataracts is the most common and treatable side effect. Reported at 83% over 5 years in the COMS study [74]. Cataracts were reported to be 11%-32% in the SRS/FSRS series and 20–62% in the PBRT series. Optic neuropathy is also a side effect that can lead to detrimental effects such as vision loss. It is reported at varying rates between 4–64% in SRS/FSRS series and 7–33% in PBRT series (Tables 1, 2 and 3).

## Conclusions

To conclude, external RT techniques are becoming increasingly common in the treatment of uveal melanoma. Although there is no prospective randomized trial comparing the treatment modalities mentioned, proton RT and SRS/FSRS oncological outcomes and eye protection rates and toxicities appear to be comparable. Although the large experience with proton therapy makes it stand out, the good oncological outcomes of SRS/FSRS state-of-the-art techniques indicate that they will become more widespread. The literature is open to further study in order to standardize the dose fractionation and treatment approach.

## Data Availability

Not applicable.

## Glossary of acronyms

ABS: American Brachytherapy Society
CK: Cyberknife
COMS: Collaborative Ocular Melanoma Study
DFS: Disease-free survival
EPB: Episcleral plaque brachytherapy
FSRS: Fractionated stereotactic radiosurgery
IMPT: Intensity-modulated spot-scanning proton therapy
Linac: Linear accelerator
MRI: Magnetic resonance image
OAR: Organs at risk
OS: Overall survival
PBRT: Proton beam radiotherapy
RBE: Relative biological effectiveness
RT: Radiotherapy
SRS: Stereotactic radiosurgery
UCSF: University of San Francisco
UM: Uveal melanoma

## References

[1] Spagnolo F, Caltabiano G, Queirolo P (2012). Uveal melanoma. Cancer Treat Rev.

[2] Shields CL, Furuta M, Thangappan A, Nagori S, Mashayekhi A, Lally DR (2009). Metastasis of uveal melanoma millimeter-by-millimeter in 8033 consecutive eyes. Arch Ophthalmol.

[3] Egan KM, Seddon JM, Glynn RJ, Gragoudas ES, Albert DM (1988). Epidemiologic aspects of uveal melanoma. Surv Ophthalmol.

[4] Kujala E, Mäkitie T, Kivelä T (2003). Very long-term prognosis of patients with malignant uveal melanoma. Invest Ophthalmol Vis Sci.

[5] Diener-West M, Reynolds SM, Agugliaro DJ, Caldwell R, Cumming K, Earle JD (2005). Development of metastatic disease after enrollment in the COMS trials for treatment of choroidal melanoma: Collaborative Ocular Melanoma Study Group Report No. 26. Arch Ophthalmol.

[6] Rantala ES, Hernberg M, Kivelä TT (2019). Overall survival after treatment for metastatic uveal melanoma: a systematic review and meta-analysis. Melanoma Res.

[7] Freton A, Chin KJ, Raut R, Tena LB, Kivelä T, Finger PT (2012). Initial PET/CT staging for choroidal melanoma: AJCC correlation and second nonocular primaries in 333 patients. Eur J Ophthalmol.

[8] Feinstein EG, Marr BP, Winston CB, Abramson DH (2010). Hepatic abnormalities identified on abdominal computed tomography at diagnosis of Uveal Melanoma. Arch Ophthalmol.

[9] Adams KS, Abramson DH, Ellsworth RM, Haik BG, Bedford M, Packer S (1988). Cobalt plaque versus enucleation for uveal melanoma: comparison of survival rates. Br J Ophthalmol.

[10] Seddon JM, Gragoudas ES, Egan KM, Glynn RJ, Howard S, Fante RG (1990). Relative survival rates after alternative therapies for uveal melanoma. Ophthalmology.

[11] Augsburger JJ, Corrêa ZM, Freire J, Brady LW (1998). Long-term survival in choroidal and ciliary body melanoma after enucleation versus plaque radiation therapy. Ophthalmology.

[12] The COMS randomized trial of iodine 125 brachytherapy for choroidal melanoma: V. Twelve-year mortality rates and prognostic factors: COMS report No. 28. Arch Ophthalmol. 2006;124(12):1684–93. 10.1001/archopht.124.12.1684 10.1001/archopht.124.12.1684 17159027

[13] Nag S, Quivey JM, Earle JD, Followill D, Fontanesi J, Finger PT (2003). The American Brachytherapy Society recommendations for brachytherapy of uveal melanomas. Int J Radiat Oncol Biol Phys.

[14] Nath R, Anderson LL, Luxton G, Weaver KA, Williamson JF, Meigooni AS (1995). Dosimetry of interstitial brachytherapy sources: recommendations of the AAPM Radiation Therapy Committee Task Group No. 43. American Association of Physicists in Medicine. Med Phys.

[15] Stallard HB (1966). Radiotherapy for malignant melanoma of the choroid. Br J Ophthalmol.

[16] Lommatzsch PK (1986). Results after beta-irradiation (106Ru/106Rh) of choroidal melanomas: 20 years' experience. Br J Ophthalmol.

[17] Finger PT, Chin KJ, Duvall G (2009). Palladium-103 ophthalmic plaque radiation therapy for choroidal melanoma: 400 treated patients. Ophthalmology.

[18] Valcárcel F, Valverde S, Cárdenes H, Cajigal C, de la Torre A, Magallón R (1994). Episcleral iridium-192 wire therapy for choroidal melanomas. Int J Radiat Oncol Biol Phys.

[19] Simpson ER, Gallie B, Laperrierre N, Beiki-Ardakani A, Kivelä T, Raivio V (2014). The American Brachytherapy Society consensus guidelines for plaque brachytherapy of uveal melanoma and retinoblastoma. Brachytherapy.

[20] Bekkering GE, Rutjes AW, Vlassov VV, Aebersold DM, von Bremen K, Jüni P (2009). The effectiveness and safety of proton radiation therapy for indications of the eye : a systematic review. Strahlenther Onkol.

[21] Wang Z, Nabhan M, Schild SE, Stafford SL, Petersen IA, Foote RL (2013). Charged particle radiation therapy for uveal melanoma: a systematic review and meta-analysis. Int J Radiat Oncol Biol Phys.

[22] Henderson MA, Shirazi H, Lo SS, Mendonca MS, Fakiris AJ, Witt TC (2006). Stereotactic radiosurgery and fractionated stereotactic radiotherapy in the treatment of uveal melanoma. Technol Cancer Res Treat.

[23] •• Kosydar S, Robertson JC, Woodfin M, Mayr NA, Sahgal A, Timmerman RD, et al. Systematic review and meta-analysis on the use of photon-based stereotactic radiosurgery versus fractionated stereotactic radiotherapy for the treatment of Uveal Melanoma. Am J Clin Oncol. 2021;44(1):32–42. 10.1097/coc.0000000000000775. **This reference is of outstanding importance because is the meta-analysis showed no difference in tumor control, survival and toxicities between stereotactic radiosurgery and fractionated stereotactic radiosurgery for uveal melanoma**. 10.1097/COC.0000000000000775 33208706

[24] Shah NV, Houston SK, Murray TG, Markoe AM (2012). Evaluation of the surgical learning curve for I-125 episcleral plaque placement for the treatment of posterior uveal melanoma: a two decade review. Clin Ophthalmol.

[25] • Trofimov AV, Aronow ME, Gragoudas ES, Keane FK, Kim IK, Shih HA, et al. A systematic comparison of dose distributions delivered in (125)I plaque brachytherapy and proton radiation therapy for ocular melanoma. Int J Radiat Oncol Biol Phys. 2023;115(2):501–10. 10.1016/j.ijrobp.2022.07.017. **This reference is of importance because it compares dose distributions of plaque brachytherapy and proton therapy**. 10.1016/j.ijrobp.2022.07.017 35878716

[26] Oxenreiter MM, Lane AM, Aronow MB, Shih H, Trofimov AV, Kim IK (2022). Proton beam irradiation of uveal melanoma involving the iris, ciliary body and anterior choroid without surgical localisation (light field). Br J Ophthalmol.

[27] Hartsell WF, Kapur R, Hartsell SOC, Sweeney P, Lopes C, Duggal A (2016). Feasibility of proton beam therapy for ocular melanoma using a novel 3D treatment planning technique. Int J Radiat Oncol Biol Physics.

[28] Bäumer C, Plaude S, Khalil DA, Geismar D, Kramer PH, Kröninger K (2021). Clinical Implementation of Proton Therapy Using Pencil-Beam Scanning Delivery Combined With Static Apertures. Front Oncol.

[29] Gragoudas ES, Goitein M, Verhey L, Munzenreider J, Suit HD, Koehler A (1980). Proton beam irradiation. An alternative to enucleation for intraocular melanomas. Ophthalmology.

[30] Egger E, Zografos L, Schalenbourg A, Beati D, Böhringer T, Chamot L (2003). Eye retention after proton beam radiotherapy for uveal melanoma. Int J Radiat Oncol Biol Phys.

[31] Damato B, Kacperek A, Chopra M, Campbell IR, Errington RD (2005). Proton beam radiotherapy of choroidal melanoma: the Liverpool-Clatterbridge experience. Int J Radiat Oncol Biol Phys.

[32] Dendale R, Lumbroso-Le Rouic L, Noel G, Feuvret L, Levy C, Delacroix S (2006). Proton beam radiotherapy for uveal melanoma: results of Curie Institut-Orsay proton therapy center (ICPO). Int J Radiat Oncol Biol Phys.

[33] Caujolle JP, Mammar H, Chamorey E, Pinon F, Herault J, Gastaud P (2010). Proton beam radiotherapy for uveal melanomas at nice teaching hospital: 16 years' experience. Int J Radiat Oncol Biol Phys.

[34] Macdonald EC, Cauchi P, Kemp EG (2011). Proton beam therapy for the treatment of uveal melanoma in Scotland. Br J Ophthalmol.

[35] Mishra KK, Daftari IK, Weinberg V, Cole T, Quivey JM, Castro JR (2013). Risk factors for neovascular glaucoma after proton beam therapy of uveal melanoma: a detailed analysis of tumor and dose-volume parameters. Int J Radiat Oncol Biol Phys.

[36] Lane AM, Kim IK, Gragoudas ES (2015). Long-term risk of melanoma-related mortality for patients with uveal melanoma treated with proton beam therapy. JAMA Ophthalmol.

[37] Seibel I, Cordini D, Rehak M, Hager A, Riechardt AI, Böker A (2015). Local recurrence after primary proton beam therapy in uveal melanoma: risk factors, retreatment approaches, and outcome. Am J Ophthalmol.

[38] Verma V, Mehta MP (2016). Clinical outcomes of proton radiotherapy for Uveal Melanoma. Clin Oncol (R Coll Radiol).

[39] Mishra KK, Quivey JM, Daftari IK, Weinberg V, Cole TB, Patel K (2015). Long-term Results of the UCSF-LBNL Randomized Trial: Charged particle with helium ion versus iodine-125 plaque therapy for choroidal and ciliary body Melanoma. Int J Radiat Oncol Biol Phys.

[40] Char DH, Kroll S, Phillips TL, Quivey JM (2002). Late radiation failures after iodine 125 brachytherapy for uveal melanoma compared with charged-particle (proton or helium ion) therapy. Ophthalmology.

[41] Gragoudas ES, Lane AM, Munzenrider J, Egan KM, Li W (2002). Long-term risk of local failure after proton therapy for choroidal/ciliary body melanoma. Trans Am Ophthalmol Soc.

[42] Gragoudas ES, Lane AM, Regan S, Li W, Judge HE, Munzenrider JE (2000). A randomized controlled trial of varying radiation doses in the treatment of Choroidal Melanoma. Arch Ophthalmol.

[43] Papakostas TD, Lane AM, Morrison M, Gragoudas ES, Kim IK (2017). Long-term outcomes after proton beam irradiation in patients with large Choroidal Melanomas. JAMA Ophthalmol.

[44] Egger E, Schalenbourg A, Zografos L, Bercher L, Boehringer T, Chamot L (2001). Maximizing local tumor control and survival after proton beam radiotherapy of uveal melanoma. Int J Radiat Oncol Biol Physics.

[45] Damato B, Kacperek A, Chopra M, Sheen MA, Campbell IR, Errington RD (2005). Proton beam radiotherapy of iris melanoma. Int J Radiat Oncol Biol Phys.

[46] Thariat J, Rahmi A, Salleron J, Mosci C, Butet B, Maschi C (2018). Proton beam therapy for Iris Melanomas in 107 patients. Ophthalmology.

[47] Gollrad J, Böker A, Vitzthum S, Besserer A, Heufelder J, Gauger U (2023). Proton therapy for 166 patients with Iris Melanoma: Side effects and oncologic outcomes. Ophthalmol Retina.

[48] Mueller AJ, Talies S, Schaller UC, Horstmann G, Wowra B, Kampik A (2000). Stereotactic radiosurgery of large uveal melanomas with the gamma-knife. Ophthalmology.

[49] Zehetmayer M, Kitz K, Menapace R, Ertl A, Heinzl H, Ruhswurm I (2000). Local tumor control and morbidity after one to three fractions of stereotactic external beam irradiation for uveal melanoma. Radiother Oncol.

[50] Simonová G, Novotný J, Liscák R, Pilbauer J (2002). Leksell gamma knife treatment of uveal melanoma. J Neurosurg.

[51] Sarici AM, Pazarli H (2013). Gamma-knife-based stereotactic radiosurgery for medium- and large-sized posterior uveal melanoma. Graefes Arch Clin Exp Ophthalmol.

[52] Dinca EB, Yianni J, Rowe J, Radatz MW, Preotiuc-Pietro D, Rundle P (2012). Survival and complications following γ knife radiosurgery or enucleation for ocular melanoma: a 20-year experience. Acta Neurochir (Wien).

[53] Wackernagel W, Holl E, Tarmann L, Mayer C, Avian A, Schneider M (2014). Local tumour control and eye preservation after gamma-knife radiosurgery of choroidal melanomas. Br J Ophthalmol.

[54] Suesskind D, Scheiderbauer J, Buchgeister M, Partsch M, Budach W, Bartz-Schmidt KU (2013). Retrospective evaluation of patients with uveal melanoma treated by stereotactic radiosurgery with and without tumor resection. JAMA Ophthalmol.

[55] Modorati GM, Dagan R, Mikkelsen LH, Andreasen S, Ferlito A, Bandello F (2020). Gamma knife radiosurgery for Uveal Melanoma: A retrospective review of clinical complications in a tertiary referral center. Ocul Oncol Pathol.

[56] Weber DC, Bogner J, Verwey J, Georg D, Dieckmann K, Escudé L (2005). Proton beam radiotherapy versus fractionated stereotactic radiotherapy for uveal melanomas: A comparative study. Int J Radiat Oncol Biol Phys.

[57] Sikuade MJ, Salvi S, Rundle PA, Errington DG, Kacperek A, Rennie IG (2015). Outcomes of treatment with stereotactic radiosurgery or proton beam therapy for choroidal melanoma. Eye (Lond).

[58] Georgopoulos M, Zehetmayer M, Ruhswurm I, Toma-Bstaendig S, Ségur-Eltz N, Sacu S (2003). Tumour regression of uveal melanoma after ruthenium-106 brachytherapy or stereotactic radiotherapy with gamma knife or linear accelerator. Ophthalmologica.

[59] van Beek JGM, Ramdas WD, Angi M, van Rij CM, Naus NC, Kacperek A (2021). Local tumour control and radiation side effects for fractionated stereotactic photon beam radiotherapy compared to proton beam radiotherapy in uveal melanoma. Radiother Oncol.

[60] Marchini G, Gerosa M, Piovan E, Pasoli A, Babighian S, Rigotti M (1996). Gamma Knife stereotactic radiosurgery for uveal melanoma: clinical results after 2 years. Stereotact Funct Neurosurg.

[61] • Eibenberger K, Dunavoelgyi R, Gleiss A, Sedova A, Georg D, Poetter R, et al. Hypofractionated stereotactic photon radiotherapy of choroidal melanoma: 20-year experience. Acta Oncol. 2021;60(2):207-14. 10.1080/0284186x.2020.1820572. **This reference is of importance because it is one of the largest series in the literature using fractionated stereotactic radiosurgery with a significant follow-up time**. 10.1080/0284186X.2020.1820572 32969745

[62] Modorati G, Miserocchi E, Galli L, Picozzi P, Rama P (2009). Gamma knife radiosurgery for uveal melanoma: 12 years of experience. Br J Ophthalmol.

[63] •• Schmelter V, Schneider F, Guenther SR, Fuerweger C, Muacevic A, Priglinger SG, et al. local recurrence in choroidal melanomas following robotic-assisted radiosurgery (CyberKnife). Ocul Oncol Pathol. 2023;8(4-6):221-9. 10.1159/000527915. **This reference is of outstanding importance because it is the largest series in the literature using stereotactic radiosurgery**. 10.1159/000527915 PMC10013483 36925728

[64] •• Yazici G, Kiratli H, Ozyigit G, Sari SY, Elmali A, Yilmaz MT, et al. Every other day stereotactic radiation therapy for the treatment of uveal melanoma decreases toxicity. Radiother Oncol. 2022;176:39-45. 10.1016/j.radonc.2022.09.010. **This reference is of outstanding importance because it is the largest series in the literature using fractionated stereotactic radiosurgery**. 10.1016/j.radonc.2022.09.010 36184996

[65] • van Beek JGM, van Rij CM, Baart SJ, Yavuzyigitoglu S, Bergmann MJ, Paridaens D, et al. Fractionated stereotactic radiotherapy for uveal melanoma: Long-term outcome and control rates. Acta Ophthalmol. 2022;100(5):511-9. 10.1111/aos.15029. **This reference is of importance because it is the study with the longest follow-up time in the literature using fractionated stereotactic radiosurgery**. 10.1111/aos.15029 PMC9544756 34529346

[66] Krema H, Somani S, Sahgal A, Xu W, Heydarian M, Payne D (2009). Stereotactic radiotherapy for treatment of juxtapapillary choroidal melanoma: 3-year follow-up. Br J Ophthalmol.

[67] Muacevic A, Nentwich M, Wowra B, Staerk S, Kampik A, Schaller U (2008). Development of a streamlined, non-invasive robotic radiosurgery method for treatment of uveal melanoma. Technol Cancer Res Treat.

[68] Eibl-Lindner K, Fürweger C, Nentwich M, Foerster P, Wowra B, Schaller U (2016). Robotic radiosurgery for the treatment of medium and large uveal melanoma. Melanoma Res.

[69] Yazici G, Kiratli H, Ozyigit G, Sari SY, Cengiz M, Tarlan B (2017). Stereotactic radiosurgery and fractionated stereotactic radiation therapy for the treatment of Uveal Melanoma. Int J Radiat Oncol Biol Phys.

[70] Muller K, Nowak PJ, Luyten GP, Marijnissen JP, de Pan C, Levendag P (2004). A modified relocatable stereotactic frame for irradiation of eye melanoma: design and evaluation of treatment accuracy. Int J Radiat Oncol Biol Phys.

[71] Castro JR, Char DH, Petti PL, Daftari IK, Quivey JM, Singh RP (1997). 15 years experience with helium ion radiotherapy for uveal melanoma. Int J Radiat Oncol Biol Phys.

[72] Langmann G, Pendl G, Müllner K, Feichtinger KH, Papaefthymiouaf G (2002). High-compared with low-dose radiosurgery for uveal melanomas. J Neurosurg.

[73] Wong AJ, Schefler AC, Teh BS (2023). Overview of late complications of radiation therapy in uveal melanoma. Chin Clin Oncol.

[74] Incidence of cataract and outcomes after cataract surgery in the first 5 years after iodine 125 brachytherapy in the Collaborative Ocular Melanoma Study: COMS Report No. 27. Ophthalmology. 2007;114(7):1363–71. 10.1016/j.ophtha.2006.10.039. 10.1016/j.ophtha.2006.10.039 17337065

